# Estimating contact-adjusted immunity levels against measles in South Korea and prospects for maintaining elimination status

**DOI:** 10.1016/j.vaccine.2019.10.040

**Published:** 2020-01-10

**Authors:** June Young Chun, Wan Beom Park, Nam Joong Kim, Eun Hwa Choi, Sebastian Funk, Myoung-don Oh

**Affiliations:** a Department of Internal Medicine, Seoul National University College of Medicine, Seoul, South Korea; b Department of Pediatrics, Seoul National University College of Medicine, Seoul, South Korea; c Centre for the Mathematical Modelling of Infectious Diseases, London School of Hygiene & Tropical Medicine, London, United Kingdom; d Department of Infectious Disease Epidemiology, London School of Hygiene & Tropical Medicine, London, United Kingdom

**Keywords:** Measles, Seroprevalence, Republic of Korea, Herd immunity

## Abstract

Measles has been reemerging in South Korea since December 2018 resulting in 185 cases by September 2019.

We calculated contact-adjusted immunity levels against measles in South Korea using national seroprevalence data in 2014, vaccination uptake rates, and an age-specific contact matrix. We further explored options to achieve a contact-adjusted immunity level of 93% for herd immunity.

The assessed contact-adjusted immunity level has increased from 86% in 2014 to 92% in 2018. Herd immunity could be achieved with immunizing 50% of susceptibles among birth cohorts 1999–2003 in 2018.

Contact-adjusted immunity levels against measles have increased recently in South Korea, although they might not yet be high enough to guarantee herd immunity.

## Highlights

• A measles outbreak is occurring in South Korea despite high vaccine coverage.
• Contact-adjusted immunity against measles is currently at 92%, increased from 86% in 2014.
• There might be an option for catch-up campaigns to achieve herd immunity.
• This is the first study to use contact patterns for understanding infectious disease outbreaks in South Korea.

## 1 Introduction

Measles, a systemic viral illness, is one of the most contagious diseases in humans. In the pre-vaccination era, approximately 2 million deaths worldwide were attributed to measles each year [1]. Thanks to a highly effective vaccine introduced in 1963 and intensive surveillance efforts, global measles incidence and mortality have gradually decreased [1]. During 2000–2015, the number of deaths due to measles dropped by 76% from 550,100 in 2000 to 134,200 in 2015 [1].

In South Korea, measles-containing vaccines (MCV) were first introduced in 1965. A single dose (MCV1) at 15 months of age was introduced in the National Immunization Program (NIP) in 1983. In 1997, NIP recommended MCV1 at 12–15 months of age and a second dose (MCV2) at 4–6 years of age with no catch-up [2]. The annual incidence of measles fell dramatically from 30,792 cases (11.6 cases per million persons) in 1962 to a low of 2 cases in 1997 [3].

However, in 2000–2001, a measles epidemic occurred that resulted in more than 50,000 cases. Following this outbreak, the Korean government announced a “Five Year Measles Elimination Program” that included a catch-up vaccination program targeting 8–16 year olds (birth cohorts March 1985 to Feb 1994, 5.8 million) and a keep-up program that required all children entering elementary school to present evidence of two-dose MCV vaccination [2], [4]. This effort reduced the annual incidence of measles to less than one per million, and, in 2006, South Korea became the first country in Western Pacific to declare elimination of measles [2].

The capacity of the measles virus to infect respiratory epithelium and release viral progeny into the airway plays a role in its high infectivity. The basic reproduction number (*R_0_*) is often cited to be between 12 and 18 for measles, although a wider range of values is possible [5]. The herd immunity level to prevent measles outbreaks requires immunity of 90 to 95% based on these values of *R_0_*, the highest of all vaccine-preventable directly transmitted diseases [5]. When taking into account differences in contact behavior, this can be achieved by ensuring that at least 85% of those aged 5 years or less are immune, and 95% of all age groups above 5 years of age [6]. Outbreaks might occur when the proportion of the population immune to measles through vaccination or prior infection drops below these percentages.

Recently in South Korea, there has been an increase in cases of measles since December 2018, and as of 28th of September 2019, a total of 185 laboratory confirmed measles cases were reported [7]. This increase occurred in spite of reported two-dose coverage over 95% since 2010 [8].

In this study, we review the changes in measles seroprevalence and vaccination uptake rate to estimate contact-adjusted immunity levels from age-specific contact patterns, which have been widely used for transmission modelling [9]. We further explore options for catch-up campaigns that might achieve sufficient levels of population immunity to maintain measles elimination.

## 2 Methods

### 2.1 Data

We used publicly available data sets of age-specific incidence of measles in South Korea [7], [10], [11]. For age-specific seroprevalence data, we used data from a nation-wide measles seroprevalence study that was conducted by KCDC in 2014 [12]. Briefly, 3050 serum samples (1000 from aged <10 years and 2050 from aged 10–50 years, birth cohorts 1964–2014) were provided by the fifth Korea National Health and Nutrition Examination Survey (KNHANES VI-1st) [13]. Measles virus-specific immunoglobulin G (IgG) antibodies were tested by an enzyme immunoassay. Equivocal results were interpreted as positive. For vaccine coverage data, we used WHO/UNICEF Estimates of National Immunization Coverage (WUENIC) [8].

### 2.2 Age-specific immunity levels

We estimated age-specific immunity levels by combining the seroprevalence data in 2014 with vaccine coverage data for birth cohorts following 2014 [12]. For those under 1 year of age at the end of 2018, we assumed that immunity levels were the same as for those under 1 year of age in 2014. For 1-year olds, we assumed that the proportion of children immune at any time compared to coverage at the end of the year would be the same in 2018 as that in 2014. For those aged 2 to 5, we assumed that their immunity was in accordance with the reported first-dose vaccination uptake between 2014 and 2017, with a vaccine efficacy of 95%. For those aged 6 or older, we used the seroprevalence levels from the KCDC study [12] shifted by 4 years and adjusted immunity levels by reported second-dose vaccination uptake (assuming that the second dose was given to those that had already received one dose), before aggregating to age groups as given by the contact data, with each single-year age group weighted according to population estimates from 2014 and 2018, respectively, when averaging them to estimate immunity in the larger age group containing multiple years of age (Table 1) [14].

**Table 1:** Proportion of the population who is immune to measles by different age groups with original seroprevalence data (2014) and elapsed time adjusted data (2018), and their contact-adjusted immunity levels in South Korea.

Age group (years)	Proportion of Immune	
	Original (2014)	Shifted (2018)
0–2	0.63	0.62
3–5	0.99	0.93
6–11	0.98	1.00
12–14	0.86	0.98
15–19	0.79	0.87
20–29	0.93	0.85
30–39	0.96	0.96
40–49	0.97	0.96
≥50	1.00	1.00

**Plain immunity**^a^	**0.92**	**0.92**

**Contact-adjusted immunity**^b^	**0.86**	**0.92**

Information based on publicly available national data conducted by Korea Centers for Disease Control in 2014 [12].

a Plain immunity was calculated by overall proportion of the population immune.

b Contact-adjusted immunity was calculated as in [18].

### 2.3 Contact matrix

**Supplementary Fig. 1:**
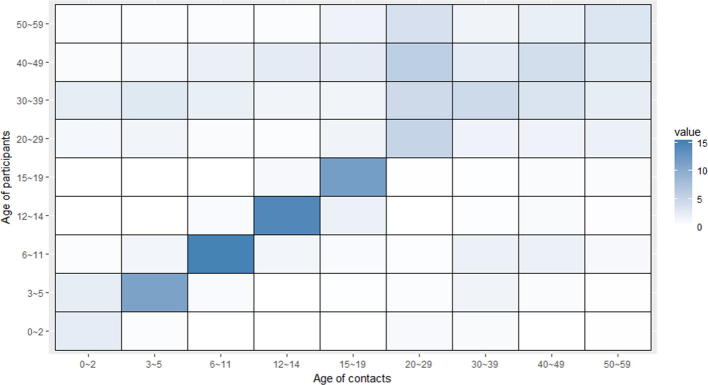
Age-specific contact patterns in Japan. It shows the reported number of contacts per day between each age groups. Adapted from Ibuka Y et al. [21].

Since social contact patterns of South Korea have not been reported, we tried to find plausible substitutes. Among East Asian countries, social mixing patterns relevant to the respiratory infections have been published for Taiwan, Japan and China [15], [16], [17]. Here, we adopted the number of contacts between age groups from a Japanese study conducted in 2011 by Ibuka Y et al. with permission (Supplementary Fig. 1) [17].

Since we did not have access to a breakdown of contacts of 20–29 year olds into smaller age groups but identified 20–24 year olds as an age group of low seropositivity, we conducted sensitivity analysis by splitting the 20–29 year old age groups in two halves, with both having half the amount of contact that 20–29 year olds have with 30–39 year olds with the other half of the age group, and the rest within their own half of the age group.

### 2.4 Plain and contact-adjusted immunity

We calculated plain immunity levels as the overall proportion of the population immune. We further combined immunity levels with contact data to calculate contact-adjusted immunity levels [18]. Contact-adjusted immunity *r’* is given by

$$r^{\text{'}}=1-\frac{R}{R_{0}}$$

where *R*/*R_0_* is the ratio of the effective reproduction number *R* to the basic reproduction number *R_0_*.

In an age-structured population, the basic reproduction number is related to the contact matrix via

$$R_{0}=\rho \left(K\right)$$

where ρ denotes the spectral radius and **K** is a matrix with elements

$$k_{\mathrm{ij}}=\phi_{\mathrm{ij}}\frac{N_{i}}{N_{j}}$$

where *ϕ_ij_* is the number of contacts that an individual in age group *i* makes with that in age group *j*, and *N_i_* is the size of age group *i*. The effective reproduction number *R* is obtained in the same way, except that the matrix **K** is multiplied with a vector of susceptibility to yield an effective matrix **K′**

$${k\text{'}}_{\mathrm{ij}}=k_{\mathrm{ij}}\left(1-r_{i}\right)$$

where *r_i_* is the proportion immune in age group *i*.

We considered 93% of contact-adjusted immunity to be the threshold level for elimination (corresponding to a basic reproductive number *R_0_* of 14.3), as in [18].

All analyses were conducted using the *R* statistical software version 3.6.1. Contact-adjusted immunity levels were calculated using the *epimixr* R package. Code and data to reproduce the analyses are available as an R package at *http://github.com/sbfnk/sk.measles*.

## 3 Results

### 3.1 Age distribution of measles in South Korea

**Fig. 1:**
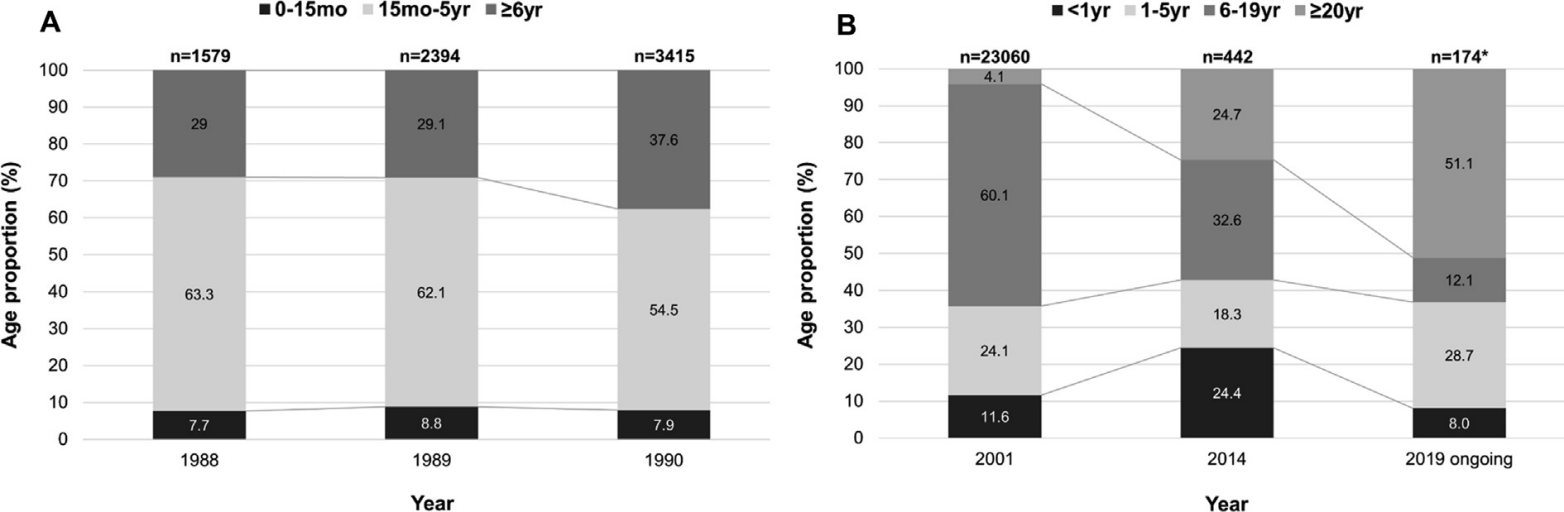
Serial age distribution of measles in South Korea before (A) and after (B) the 2000–2001 outbreak. * denotes that the number includes suspected number of measles cases, not only confirmed cases, since the outbreak is still ongoing.

Age distribution of measles cases according to years is shown in Fig. 1. The proportion of all measles cases in those aged 5 years or less was 71% in 1989, 35.7% in 2001, and 42.7% in 2014, while the proportion of cases in those aged 20 years or more was 4.1% in 2001, and 24.7% in 2014.

### 3.2 Contact-adjusted immunity

The contact-adjusted immunity level estimated based on the 2014 seroprevalence data was 86%, corresponding to an effective reproduction number of 1.7–2.5 if the basic reproduction number *R_0_* is assumed to be in the typical range of 12–18 [5]. The plain population-averaged immunity level ignoring social mixing patterns was 92%. Projecting these immunity levels to the end of 2018 resulted in estimated 92% of contact-adjusted immunity, corresponding to an effective reproduction number of 1.0–1.4 if the basic reproduction number *R_0_* is 12–18, and 92% plain immunity (Table 1). Sensitivity analysis conducted by splitting the 20–29 year age group in two halves yielded the same level of immunity.

### 3.3 Scenarios

**Fig. 2:**
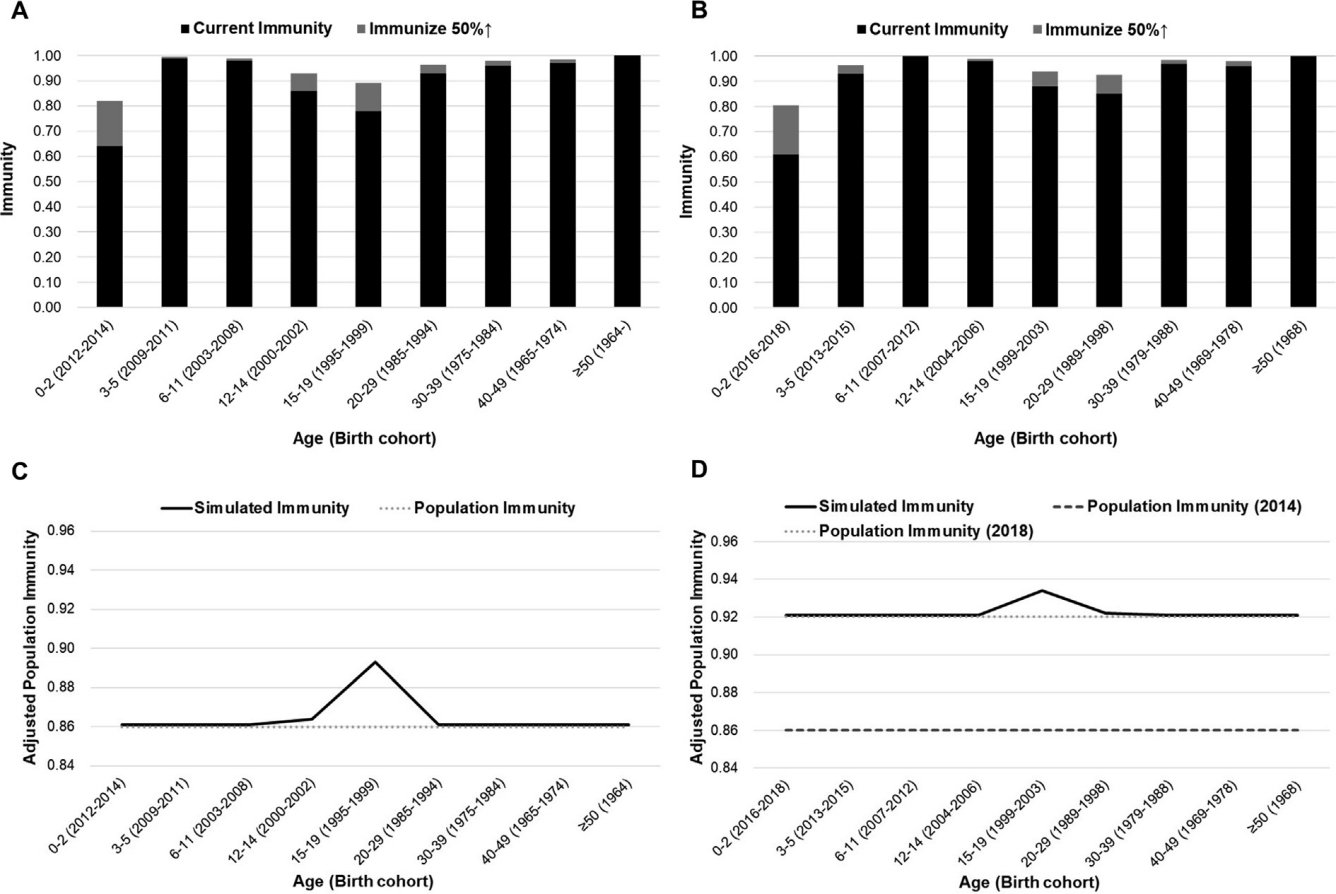
Scenario modelling for contact-adjusted immunity through raising immunity of each age group by 50% in perspective of 2014 (A) and 2018 (B). The most dramatic effect was achieved in the 15–19 year olds group both in 2014 (C) and 2018 (D), compared to estimated population immunity of 2014 (86%) and 2018 (92%).

We tested alternative scenarios where we changed immunity levels of each age group, to test the effect on contact-adjusted immunity that could be achieved in vaccination campaigns. Immunizing 50% of susceptibles in each age groups, the biggest effect was achieved in the 1519 year olds group (birth cohorts 1999–2003), which led to adjusted immunity level of 93% from the perspective of 2018 (Fig. 2D). This is in contrast to the situation in 2014, when the greatest effect was also achieved in 15–19 year olds (birth cohorts 1995–1999), but this would have raised contact-adjusted immunity levels only to 89% (Fig. 2C).

## 4 Discussion

The seroprevalence data collected in 2014 indicated that there was an immunity gap in birth cohorts 1993–2000 [12]. This immunity gap has been reported in two serological studies conducted by KCDC [12], [19]. It is in line with currently observed age distribution of measles, which is mainly concentrated in those aged over 20. The low contact-adjusted population immunity of 86% in 2014 is attributable to this gap generation, since those age groups were at 14–21 year-old school age whose contact numbers are greater than most other age groups. On the other hand, projecting immunity levels to 2018 using vaccination coverage data leads to 92% of contact-adjusted population immunity. It seems like the susceptible cohort has grown out of the school age and, consequently, the risk of a large outbreak has been reduced. A caveat is that we do not have a breakdown in contacts between 20 and 24 (where most susceptibles are) and 25–29 year olds. While we did sensitivity analysis to account for this, yielding no different results, we might still underestimate assortative contacts of 20–24 year olds. According to national index data, percentage of students going to college reaches 70% in South Korea, and this could cause higher contact rates among those between 20 and 24 years of age [20].

While contact-adjusted immunity is increasing as susceptibles are ageing out of the most contact-intense settings (especially schools) and large outbreaks are thus less likely, the immunity gap that continues to exist in certain birth cohorts still raise concerns. If individuals in this age group become parents and get infected with measles virus, then they could transmit the virus to their susceptible infants.

To estimate the spread of respiratory transmissible disease such as measles or influenza [17], understanding contact patterns between individuals is important. Changes in contact patterns might determine the transmission dynamic of the pathogen, such as whether it will become epidemic or contained in an endemic level. Infectious disease modelling based on these observed contact patterns has repeatedly been shown to predict infectious disease dynamics better than those based on random mixing [9], [18].

Although this study provides insightful analysis for the current measles outbreak in South Korea, there are some limitations. First, the employed contact matrix is not of South Korea, but of a nearby country, Japan. East Asian contact matrices from Taiwan, Japan and China have shown similar contact patterns of age-assortative mixing, leading us to assume South Korea might be comparable [15], [16], [17]. Besides, the Japanese contact matrix was based on a retrospective survey, and as such may suffer from recall bias. It remains an open question if diary-based studies of contact are sufficiently precise to warrant conducting country-specific mixing studies. If yes, establishing a South Korean contact matrix may improve the accuracy of our analysis. Second, to gain up-to-date population immunity levels from the seroprevalence study in 2014, we projected immunity levels forward to 2018 with observed vaccination rates. However, such projections are generally not as accurate as observed national seroprevalence levels, as assumptions need to be made on how exactly coverage translates to immunity levels in different age groups. Further, we assumed that vaccine-induced immunity against measles did not wane over time, but a recent study suggested waning immunity against measles [12]. More seroprevalence studies would be needed to fully quantify the risk of measles outbreaks in South Korea. Third, we interpreted equivocal results of measles IgG as positive. If these were susceptible to measles, population immunity would be lower (69% contact-adjusted immunity in 2014, 83% in 2018).

Here, we presented contact-adjusted population immunity against measles in South Korea. Furthermore, we propose target age groups for catch-up campaigns to guarantee herd immunity.

## 5 Ethics approval

All the data used in this study was publicly available, and therefore were regarded exempt from institutional review board assessment.

## Funding

Dr. Sebastian Funk was supported by a Wellcome Trust Senior Research Fellowship (210758/Z/18/Z).

## Declaration of Competing Interest

The authors declare that they have no known competing financial interests or personal relationships that could have appeared to influence the work reported in this paper.
